# Development of a latex agglutination test based on VH antibody fragment for detection of *Streptococcus suis* serotype 2

**DOI:** 10.1371/journal.pone.0299691

**Published:** 2024-04-03

**Authors:** Kiratika Chidkoksung, Nattihda Parakasikron, Suphachai Nuanualsuwan, Kannika Khantasup

**Affiliations:** 1 The Medical Microbiology Program, Graduate School, Chulalongkorn University, Bangkok, Thailand; 2 Department of Veterinary Public Health, Faculty of Veterinary Sciences, Chulalongkorn University, Bangkok, Thailand; 3 Food Risk Hub, Research Unit of Chulalongkorn University, Bangkok, Thailand; 4 Department of Biochemistry and Microbiology, Faculty of Pharmaceutical Sciences, Chulalongkorn University, Bangkok, Thailand; Tokyo University of Pharmacy and Life Sciences: Tokyo Yakka Daigaku, JAPAN

## Abstract

*Streptococcus suis* serotype 2 (SS2) is an important porcine pathogen that causes diseases in both swine and human. For rapid SS2 identification, a novel latex agglutination test (LAT) based on heavy-chain variable domain antibody (VH) was developed. Firstly, the soluble 47B3 VH antibody fragment from a phage display library, in which cysteine residues were engineered at the C-terminus, was expressed in *Escherichia coli*. The purified protein was then gently reduced to form monomeric soluble 47B3 VH subsequently used to coat with latex beads by means of site-specific conjugation. The resulting VH-coated beads gave a good agglutination reaction with SS2. The LAT was able to distinguish *S*. *suis* serotype 2 from serotype 1/2, which shares some common sugar residues, and showed no cross-reaction with other serotypes of *S*. *suis* or other related bacteria. The detection sensitivity was found to be as high as 1.85x10^6^ cells. The LAT was stable at 4°C for at least six months without loss of activity. To the best of our knowledge, this is the first LAT based on a VH antibody fragment that can be considered as an alternative for conventional antibody-based LAT where VHs are the most favored recombinant antibody.

## Introduction

During the past few years, the number of reported SS2 infections in humans has increased significantly. Most cases originate in Southeast Asia, where pig-rearing and eating uncooked pork is common [1–3]. Increased awareness and improved diagnostics have contributed to decrease the death rate from SS2 infections. To date, *S*. *suis* can be divided into 29 serotypes based on genetic analysis [4, 5]. Some previously recognized serotypes; serotypes 20, 22, 26, 32, 33, and 34 were classified as other *Streptococcus* species [6–10]. Typically, diagnosis of SS2 infections and serotyping can be achieved by direct culture and biochemical tests followed by multiplex PCR [1, 11], but these processes take several days to complete. Sometimes the biochemical tests show a false-positive with other *Streptococcus* species or it is often misidentified, resulting in the infection going undiagnosed [12]. Meanwhile, serological typing with CPS-specific antibodies also showed cross-reaction between serotypes 2, 1/2, as they share some common sugar residues [13]. Thus, loop-mediated isothermal amplification (LAMP) assay, mismatch amplification mutation assays (MAMA-PCR), PCR-restriction fragment length polymorphism (PCR-RFLP), and other molecular based techniques have been developed to differentiate between *S*. *suis* serotype 2 from 1/2 based on a single nucleotide polymorphism (SNP) in the cpsK gene; however, these methods required high-cost, specific tools and an examination specialist for the examination [14–18].

The latex agglutination test (LAT) is an alternative way to diagnose and serotype SS2 infection. It is easy, rapid, and can be used to test directly from a colony after culturing or for direct antigen detection in specimens. The method does not require specific laboratory tools for its operation, which makes it suitable for use in general hospital laboratories or health stations that encounter a high risk of SS2 infected patients and limited resources.

In recent years, heavy-chain variable domains (VH), also called single domain antibodies, have become one of the more attractive recombinant antibody fragments [19, 20]. The utility of VH for a broad range of applications, ranging from therapy as cancer targeting and diagnosis as bacterial contamination detection, has been demonstrated [21–23]. The VH antibody fragment can be derived from the heavy-chain variable domains of antibodies from camels, cartilaginous fishes, or human [24, 25]. Compared to full-length antibodies, the VH is made of one polypeptide and so they can recognize more epitopes, particularly some “hidden” or cryptic epitopes, and they can be easily produced in bacteria or yeast [26–28].

Recently, we successfully isolated the phage clone 47B3 VH from a human VH phage library, selected as that with the highest specific binding activity to SS2 with no cross-reactivity with SS1/2, a serotype that shares a common antigenic epitope with serotype 2 [29]. It is likely that 47B3 VH can bind to cryptic epitopes that can differentiate among these subtypes.

Conjugation with latex beads have endowed versatile functions to antibodies, resulting in antibody conjugates that are capable of being used for the LAT. To avoid interference with the antibody target binding, site specific conjugation methods have been developed with the aim of directing the conjugation to a specific location on the antibody [30]. In this paper, we describe the development of a LAT using 47B3 VH, which is directed against the capsule polysaccharide antigen of SS2. Phage clone 47B3 VH was converted to soluble 47B3 VH. The specific unpaired C-terminal cysteine on VH was used to site-specifically conjugate the VH to maleimide linker latex beads. The specificity of the derived LAT, based upon the agglutination reactivity of the VH-coated latex particles, was evaluated with different concentrations of SS2 cells.

## Materials and methods

### Construction and expression of soluble 47B3 VH

The phage clone 47B3 VH, which showed a specific binding to the capsule polysaccharide of SS2, was selected to express as a soluble VH antibody. The coding sequence described in a previous report was synthetically prepared by Invitrogen company (Genscript, USA) using the codon preference of *E*. *coli* and the amber stop codon was replaced with glutamine [20]. The sequences, including a 5’ restriction site for *Nco*I, 6 x His tag, unpaired cysteine at the C-terminus, and a 3’ restriction site for *Not*I were added in the sequence, respectively. The *Nco*I-*Not*I fragment containing the coding sequence was excised from the derived 47B3-pUC57 plasmid and ligated into the pET28b+ vector (Genscript, USA) that had likewise been digested with *Nco*I and *Not*I enzymes. The resulting recombinant 47B3 VH plasmid was transformed into competent *E*. *coli* SHuffle^®^ T7 cells (New England Biolabs, Massachusetts, USA). The recombinant 47B3 VH plasmid-transformed *E*. *coli* SHuffle was grown in fresh Luria-Bertani broth supplemented with 50 μg/mL kanamycin and cultured at 30°C to an optical density at 600 nm of 0.6–0.8. Next, the expression of the recombinant protein was induced by adding 0.5 mM isopropyl β-d-1-thiogalactopyranoside (IPTG) and culturing for 20 h at 30°C. The cells were harvested by centrifugation at 6,000 x g for 15 min and stored at -80°C until used for protein extraction.

For protein extraction, the pellet was resuspended in lysis buffer [150 mM NaCl, 1% (w/v) Triton x-100, 50 mM Tris-HCl, and 20 mM imidazole] and incubated on ice for 15 min before being lysed by sonication (10 s pulse cycle at 35% amplitude) on ice. The crude lysate was centrifuged at 6,000 x g for 15 min and the pellet and supernatant were separately harvested. The clear crude lysate (supernatant) was purified using nickel-nitrilotriacetic acid (Ni-NTAA) agarose column chromatography (ACC; Cytiva, Uppsala, Sweden). The lysate was loaded onto a pre-equilibrated Ni-NTAA column using binding buffer that contained 20 mM imidazole. After that, the column was washed in washing buffer containing 40 mM imidazole and then the bound soluble 47B3 VH was eluted from the column using eluting buffer containing 400 mM imidazole. The eluted fractions were screened for protein by 15% (w/v) sodium dodecyl sulphate-polyacrylamide gel electrophoresis (SDS-PAGE) under a reducing condition and western blot, was detected by mouse anti-His-tag AP conjugate and BCIP/NBT AP substrate (Surmodics IVD, Inc., Eden Prairie, USA).

### Characterization of soluble 47B3 VH

To determine the cross-reactivity of soluble 47B3 VH, ATCC reference strain of *S*. *suis* serotypes 2, 1/2, 1, 5, 6, 14, 16, and 24, plus three serotype 2 human clinical isolates were used [22]. Furthermore, other bacteria that can be found in the bloodstream of sepsis patients, such as *Streptococcus pyogenes* (*S*. *pyogenes*), *Staphylococcus aureus (S*. *aureus)*, *Escherichia coli (E*. *coli)*, *Pseudomonas aeruginosa (P*. *aeruginosa)*, and *Enterobacter aerogenes (E*. *aerogenes)* ATCC reference strain were also used to test for cross-reactivity by ELISA. Bacterial cells (1.5 x 10^7^ cells/well) were coated at 4°C overnight. The plate was washed with phosphate buffered saline pH 7.4 (PBS) and non-specific binding was blocked with PBS containing 2% (w/v) powdered milk (MPBS) for 1 h at 37°C. After washing with PBS, 50 μL of soluble 47B3 VH in two-fold dilutions from 20 μg/mL to 0.625 μg/mL diluted in MPBS were added. The plate was further incubated at 37°C for 1 h, then washed with PBS containing 0.01% (v/v) Tween-20 (0.01% PBST). The cell-bound VH were sequentially incubated with anti-his tag antibody (1:2,000; Cell Signaling Technology, Massachusetts, USA) in 1% MPBS and horseradish peroxidase (HRP)-conjugated sheep anti-mouse IgG (1:2,000; GE Healthcare, UK) at 37°C for 1 h. The plate was subsequently washed five times with 0.01% PBST. The HRP activity was determined using the TMB-substrate (Surmodics IVD, Inc., Eden Prairie, USA) and monitoring the color change at 450 nm (A_450_) using a CALIOstar Microplate reader (BMG LABTECH, Ortenberg, Germany).

### Preparation of 47B3 VH for site-specific conjugation

To obtain the monomeric 47B3 VH-SH for site specific conjugation, the soluble 47B3 VH was first reduced with different molar ratios of VH: dithiothreitol (DTT) at 1:40, 1:60, 1:80, and 1:100 and incubated at 37 ˚C for 1 h. After the respective incubation time, the reduced antibody was mixed with loading dyes and evaluated for the presence of the dimer and monomer forms by non-reducing 15% (w/v) SDS-PAGE and visualized by coomassie brilliant blue g-250.

After the reduction process, the excess DTT was removed from the reduced antibody using a vivaspin 500 3-kDa cutoff centricon filter (Cytiva, Uppsala, Sweden). The binding ability of the reduced soluble 47B3 VH with SS2 was tested by ELISA. The overnight culture of SS2 (1.5x 10^7^ cells/well was coated in ELISA wells. The plate was washed five times with PBS. Non-specific binding was blocked with MPBS for 1 h at 37°C. After washing five times with PBS, 5 μg/mL of non-reduced or reduced 47B3 VH (at VH: DTT mole ratios of 1:40 and 1:60) were added and the plate was incubated at 37°C for 1 h and then washed five times with 0.05% PBST. The cell-bound Abs were detected using a 1:2000 dilution of sheep anti-mouse IgG-HRP conjugate (GE Healthcare, UK) in MPBS. Unbound antibodies were removed by washing with 0.05% PBST. The HRP activity was detected as described in the cross-reactivity test.

### Preparation of VH-coated beads

Firstly, to create the maleimide-linked latex beads, 25 μL of 5% (w/v) 0.8-μm amino polystyrene latex beads (Bangs Laboratories, Inc., Indiana, USA) were washed three times with PBS (pH 7.2). The latex bead suspension was then mixed with (sulfosuccinimidyl 4-(N-maleimidomethyl) cyclohexane-1-carboxylate (Sulfo-SMCC) linker (Thermo Fisher Scientific, Massachusetts, USA) at a bead: Sulfo-SMCC ratio of 1:6 and rotated on a rotary shaker for 30 mins at room temperature (RT). After incubation, the beads were washed three times with PBS to remove excess sulfo-SMCC and then resuspended in 25 μL of PBS. Next, 25 μg of reduced soluble 47B3 VH was combined with the freshly made maleimide-linked latex bead preparation and rotated on a rotary shaker for 1 h at RT to allow conjugation. After conjugation, the 47B3 VH-coated beads were washed five times with PBS to remove the excess VH and then resuspended in PBS containing 0.1% (w/v) bovine serum albumen and 0.1% (w/v) sodium azide to give a 0.8% (w/v) suspension of VH-coated bead particles.

### The LAT

The LATs were conducted on a glass slide with black background. On each slide, 25 μL of 47B3 VH-coated bead suspension and 25 μL of SS2 suspension (1 colony in 150 μL PBS) were combined thoroughly, and then the glass slide was manually rocked from side to side for up to 5 min to provoke the agglutination reaction. Non-coated beads were likewise mixed with SS2 suspensions as a negative control. In addition, *S*. *suis* serotypes 1/2, 1, 5, 6, 14, 16, and 24 as well as other bacteria that can be found in the bloodstream of sepsis patients, such as *S*. *pyogenes*, *S*. *aureus*, *E*. *coli*, *P aeruginosa*, and *E*. *aerogenes* were also used as above to test for cross-reactivity. Moreover, several dilutions of an overnight-grown culture of SS2 were applied as above in the LAT to ascertain the sensitivity of the test. The agglutination results were evaluated by visual inspection macroscopically with the naked eye and scored as follows: (i) +++, a strong and clear agglutination appeared within 1 min; (ii) ++, visible agglutinated clumps appeared after a delay of 1–3 min; (iii) +, the latex suspensions were converted to visible white clumps within 5 min; and (iv) -, no agglutination was observed during 5 min.

For stability evaluation, the 47B3 VH-coated beads were kept at 4°C and agglutination tests were performed against SS2 and *E*. *coli*, as the negative control, every week for 6 months.

### Statistical analysis

Data are expressed as the mean ± one standard deviation (SD). Statistical analysis was performed using the SPSS version 22.0 software (SPSS Inc., Chicago, IL, USA).

## Results

### Construction and expression of soluble 47B3 VH

A single transformed *E*. *coli* colony containing the recombinant 47B3 VH plasmid was selected for expression of the soluble VH in subsequent experiments. To optimize the condition for inducing protein expression, recombinant 47B3 VH in the *E*. *coli* SHuffle^®^ T7 was induced by different concentrations of IPTG (0.1, 0.5, and 1 mM) at 30°C for 20 h, lysed by sonication, and centrifuged. The expression of soluble 47B3 VH in the cell pellet and supernatant was analyzed by SDS-PAGE. The recombinant protein with a size of approximately 14 kDa was successfully expressed and was found at the highest expression level in the supernatant fraction following induction with 0.5 mM IPTG.

**Fig 1:**
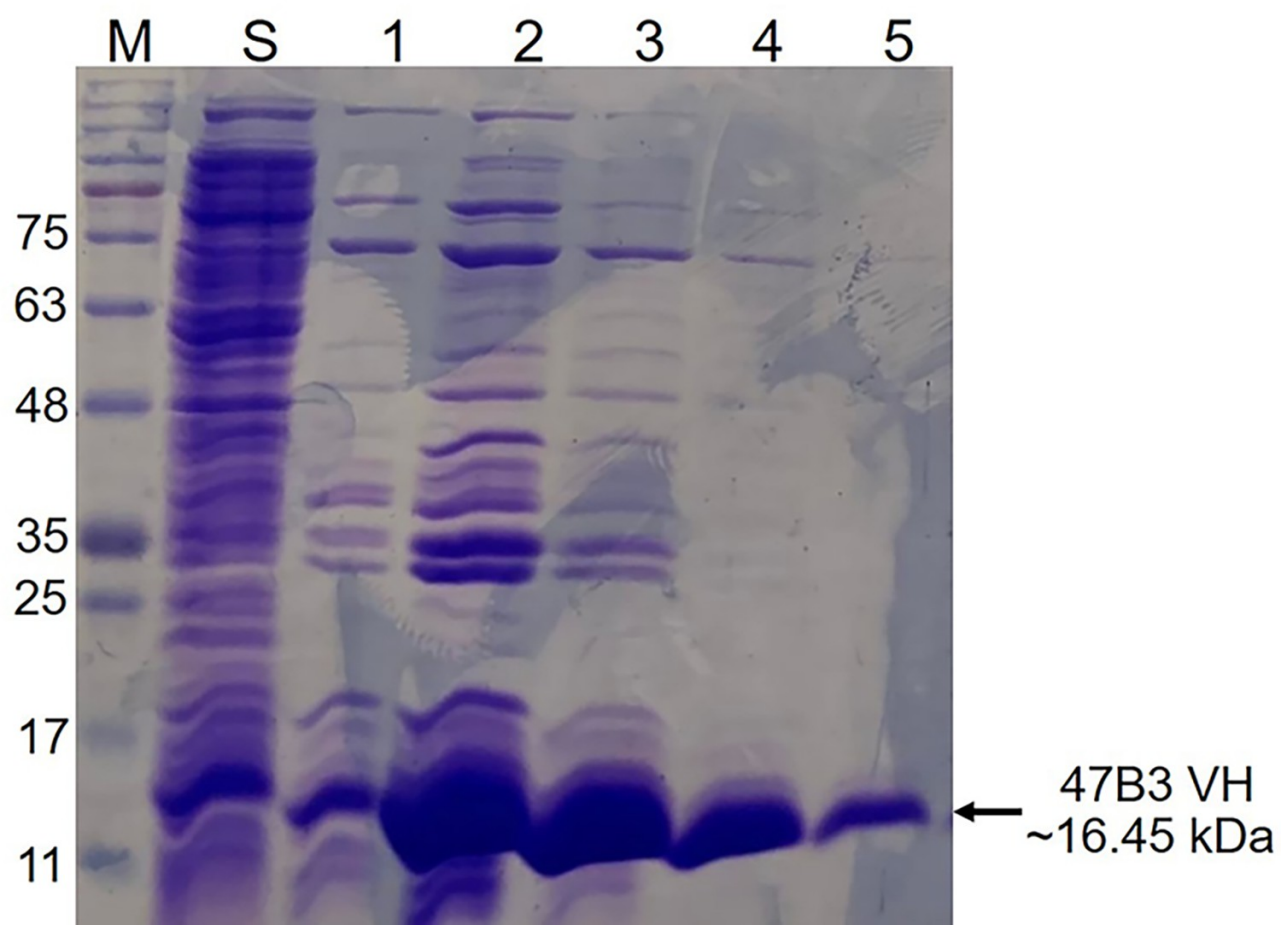
Representative SDS-PAGE analysis showing the purification of the recombinant soluble 47B3 VH protein. M: Marker; S: soluble 47B3 VH in the supernatant before purification; 1–5: elution fractions 1 to 5 of soluble 47B3 VH after purification.

Given the recombinant soluble 47B3 VH had a 6 x his tag sequence, it was further purified from the induced supernatant using Ni-NTAA-ACC. The eluted fractions were screened by SDS-PAGE analysis (Fig 1). The eluted fractions containing the soluble 47B3 VH were pooled and the total protein concentration determined using a Bradford protein assay, revealing a net purified protein yield of approximately 1.69 mg/L of culture.

### Characterization of soluble 47B3 VH

**Fig 2:**
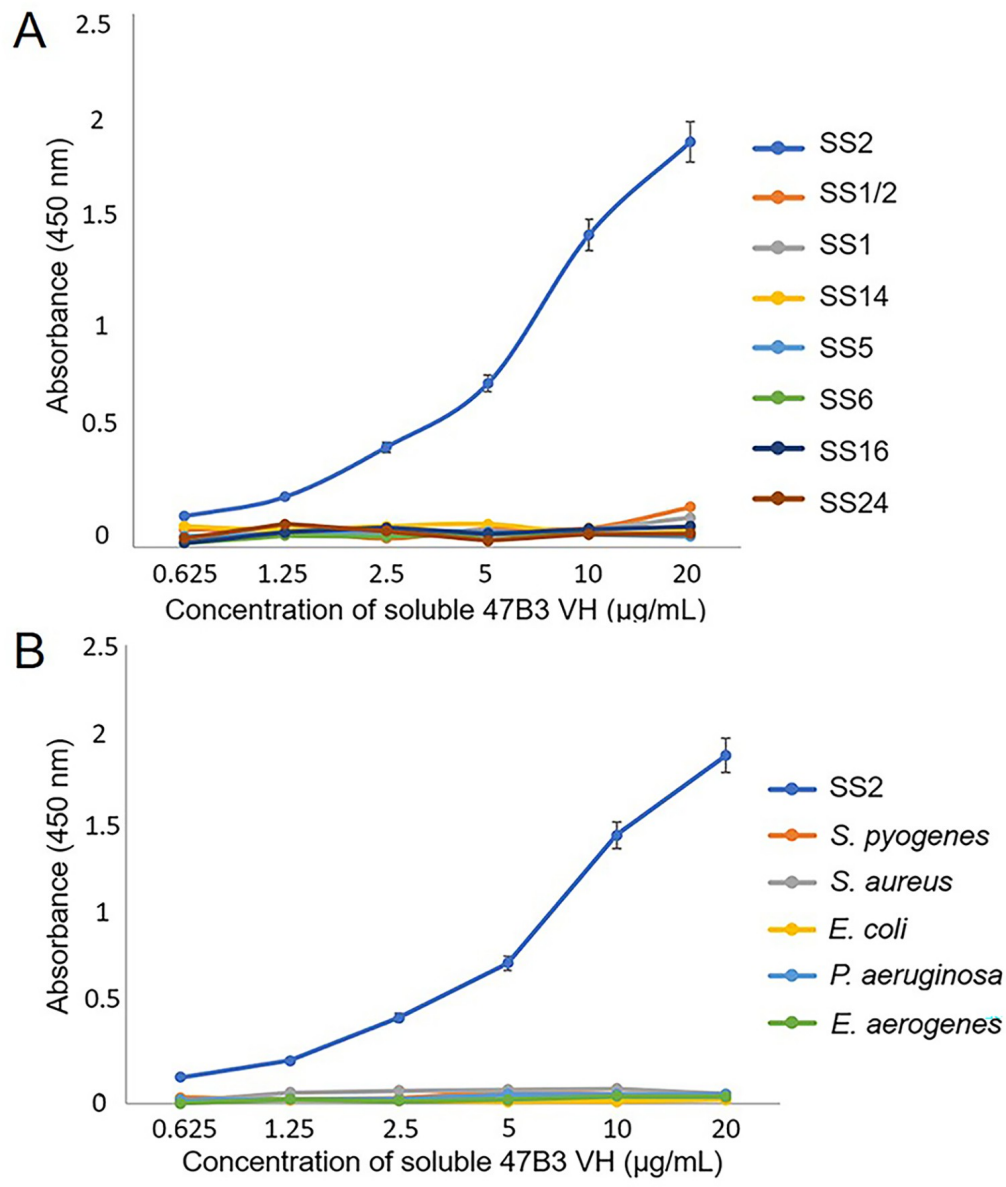
The specificity and cross-reactivity of soluble 47B3 VH against tested bacteria. (A) *S*. *suis* serotypes 2, 1/2, 1, 14, 5, 6, 16, and 24, and (B) *S*. *pyogenes*, *S*. *aureus*, *E*. *coli*, *P*. *aeruginosa*, and *E*. *aerogenes*, as tested by ELISA. Bars represent the mean of three replicate wells and error bars indicate the SD of the mean (n = 3).

The cross-reactivity profiles of the soluble 47B3 VH were determined against other bacteria that could cause a false-positive when testing samples suspected to be SS2. The tested bacteria were divided in two groups. In addition to SS2 in each group, the first group was comprised of *S*. *suis* serotypes 1/2, 1, 5, 6, 14, 16, and 24, which have been occasionally reported of human cases. The second group was comprised of some of the bacteria that can be found in the bloodstream in sepsis patients, such as *S*. *pyogenes*, *S*. *aureus*, *E*. *coli*, *P*. *aeruginosa*, and *E*. *aerogenes*, plus SS2 for direct comparison. In the ELISA results, soluble 47B3 VH had no cross-reactivity with any of these other tested *S*. *suis* serotypes (group 1) or bacteria (group 2) but was specific for SS2 (Fig 2).

**Fig 3:**
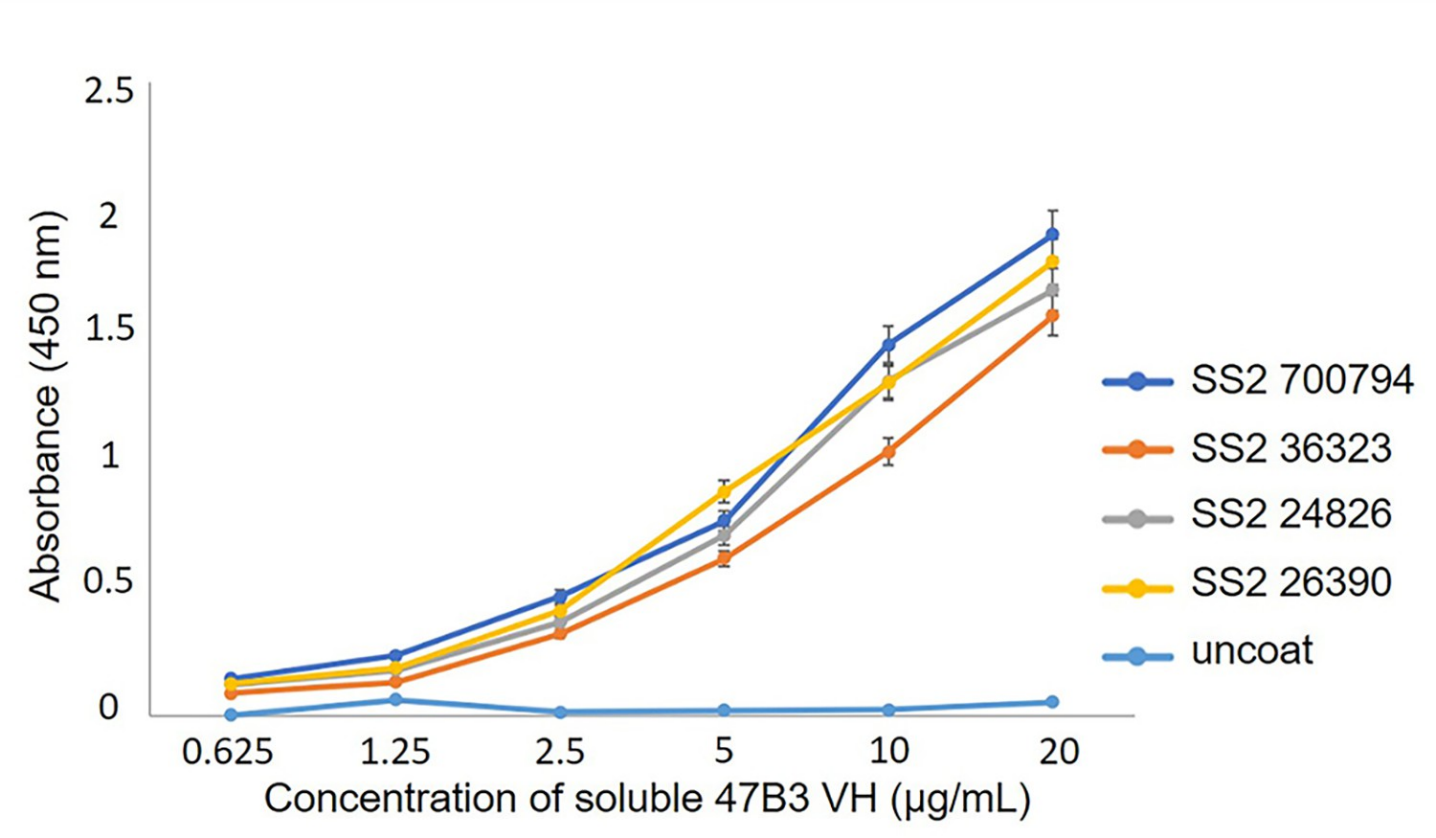
The specificity of soluble 47B3 VH against SS2 and three serotype 2 human clinical isolates, as tested by ELISA. Bars represent the mean of three replicate wells and error bars indicate the standard deviation of the mean (n = 3).

Moreover, the soluble 47B3 VH had a high binding activity with all three tested SS2 human clinical isolates in a specific and dose-dependent manner (Fig 3).

### Preparation of 47B3 VH for site-specific conjugation

**Fig 4:**
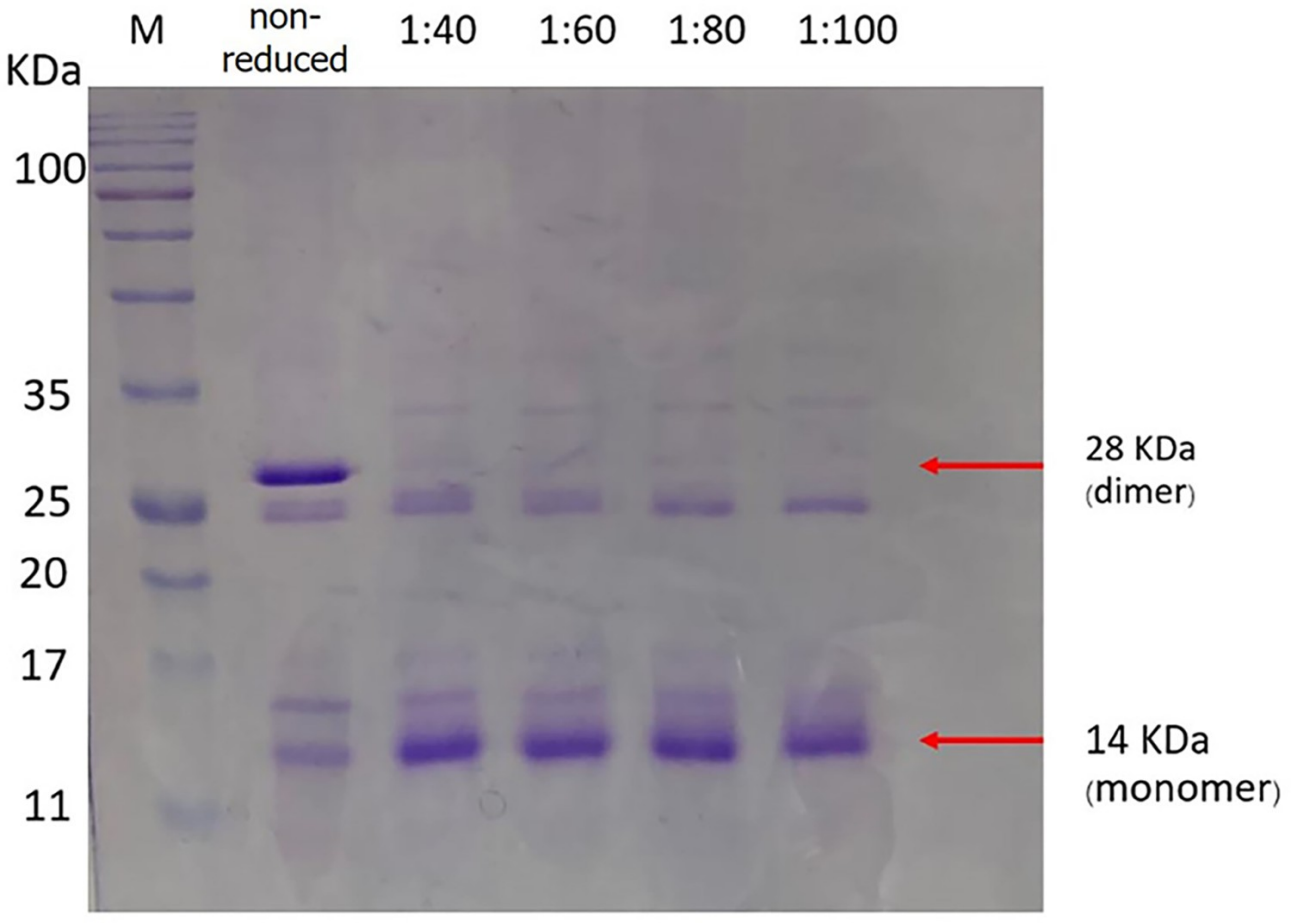
Representative non-reducing SDS-PAGE analysis showing the dimer and monomer forms of 47B3 VH after reduction with different VH: DTT molar ratios. Lane 1: protein MW marker; Lane 2: non-reduced soluble VH fraction; Lanes 3–6: soluble 47B3 VH fraction reduced with VH: DTT molar ratios of 1:40, 1:60, 1:80, and 1:100, respectively.

Soluble 47B3 VH was reduced with different molar ratios of VH: DTT (1:40, 1:60, 1:80, and 1:100) and then visualized for monomer or dimers using non-reducing SDS-PAGE (Fig 4). Following reduction, there was a clear decrease in the intensity of the dimer band (at around 28 kDa) and an increase in the intensity of the monomer band (at around 14 kDa) at all VH: DTT ratios. Thus, VH: DTT molar ratios between 1:40 to 1:100 were appropriate to reduce the 47B3 to give 98% in the monomer form determined in non-reducing SDS-PAGE. Since VH: DTT molar ratios above 1:60 did not show any reduced dimeric and increased monomeric VH, compared to that at a 1:60 ratio, but could risk reducing intradomain disulfide bonds and compromise the antigen binding capacity, VH: DTT molar ratios of 1:40 and 1:60 were chosen to reduce the 47B3 VH and test for antibody activity after reduction.

**Fig 5:**
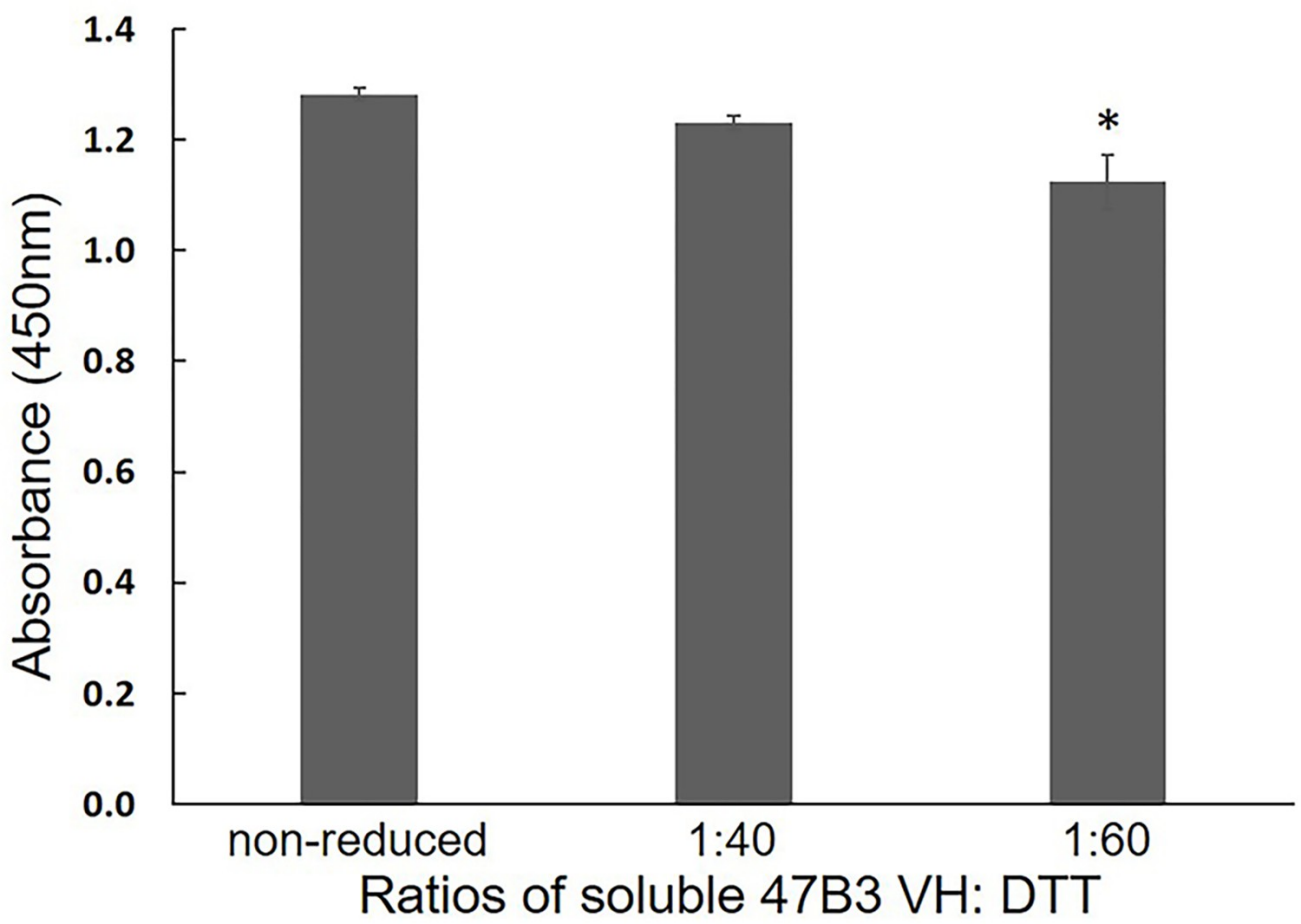
The SS2 binding activity of soluble 47B3 VH after being reduced at various VH: DTT molar ratios, as tested by ELISA. Data are shown as the mean ± 1SD (n = 3). *P < 0.05 compared to the non-reduced 47B3 VH.

Whether the soluble 47B3 VH still maintained its binding ability to SS2 after DTT reduction was evaluated by ELISA. The binding activity of the reduced 47B3 VH at a VH: DTT molar ratio of 1:40 was not significantly reduced, whereas it was at a 1:60 ratio (Fig 5). Therefore, we chose a VH: DTT molar ratio of 1:40 as optimal for the preparation of the monomeric 47B3 VH-SH for site directed conjugation.

### The LAT

Following the immobilization of the 47B3 VH on the latex beads, the functionality of the 47B3 VH-coated beads was verified by combining equal volumes (25 μL) of the test bacterial suspension with the 47B3 VH-coated beads on a glass slide. The relative strength of the agglutination was graded from +++ to + and -, depending on the strength of agglutination, as described in methods.

**Fig 6:**
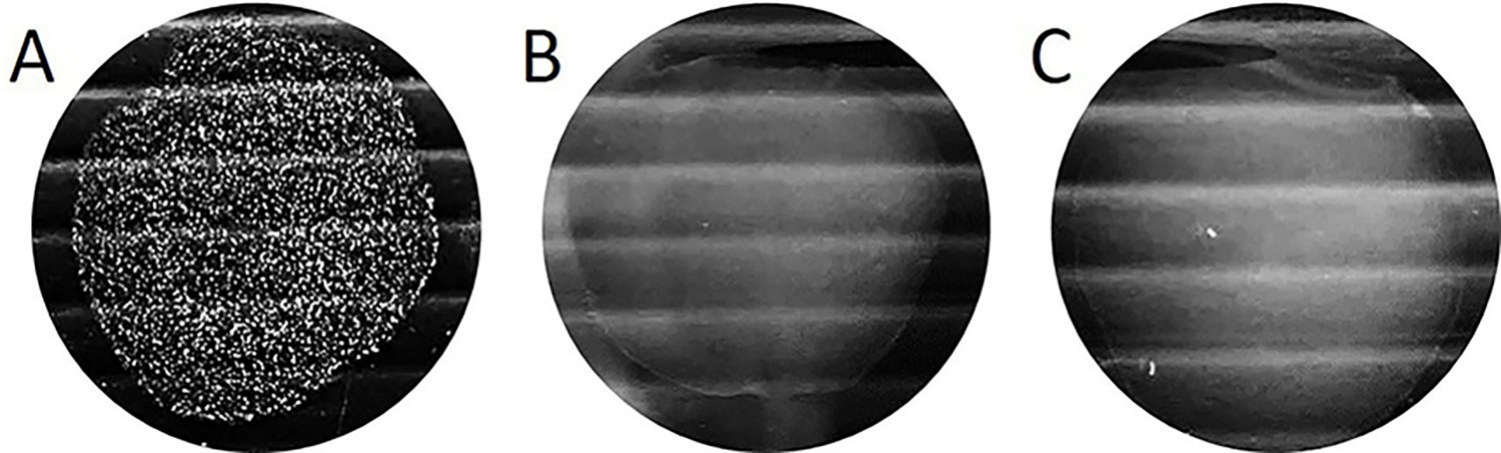
Representative images of the LAT using 47B3 VH-coated beads mixed with tested bacteria. (A) SS2 cells and (B) SS1/2 cells; and (C) non-coated beads mixed with SS2 cells (negative control).

The agglutination test showed that the newly developed LAT had a satisfactory agglutination result with SS2 with the strength of agglutination of +++. There was no cross reaction with SS1/2, which is important as these two serotypes cannot be differentiated by multiplex PCR. Rather, it gave a strong (+++) and negative (-) reaction to serotype 2 and 1/2, respectively (Fig 6). The non-coated beads gave a completely negative agglutination reaction with SS2.

**Fig 7:**
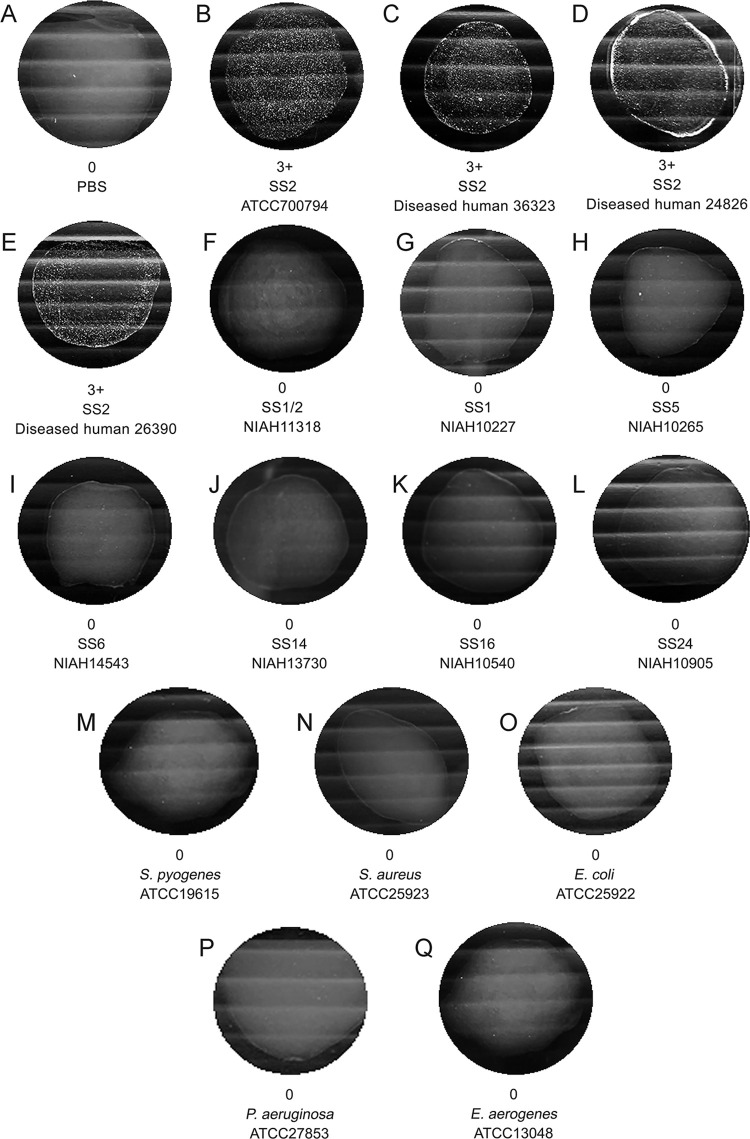
Representative images of the LAT using 47B3 VH-coated beads mixed with suspensions of different bacterial serotypes or species to test for cross-reactivity.

In addition, a strong positive agglutination reaction (+++) was seen with all three of the tested SS2 human clinical isolates, while no agglutination (-) was seen for all other tested *S*. *suis* serotypes, which were those that occasionally cause infections in human, and all the other tested bacteria that can be found in the bloodstream of sepsis patients, were also negative (Fig 7).

**Fig 8:**
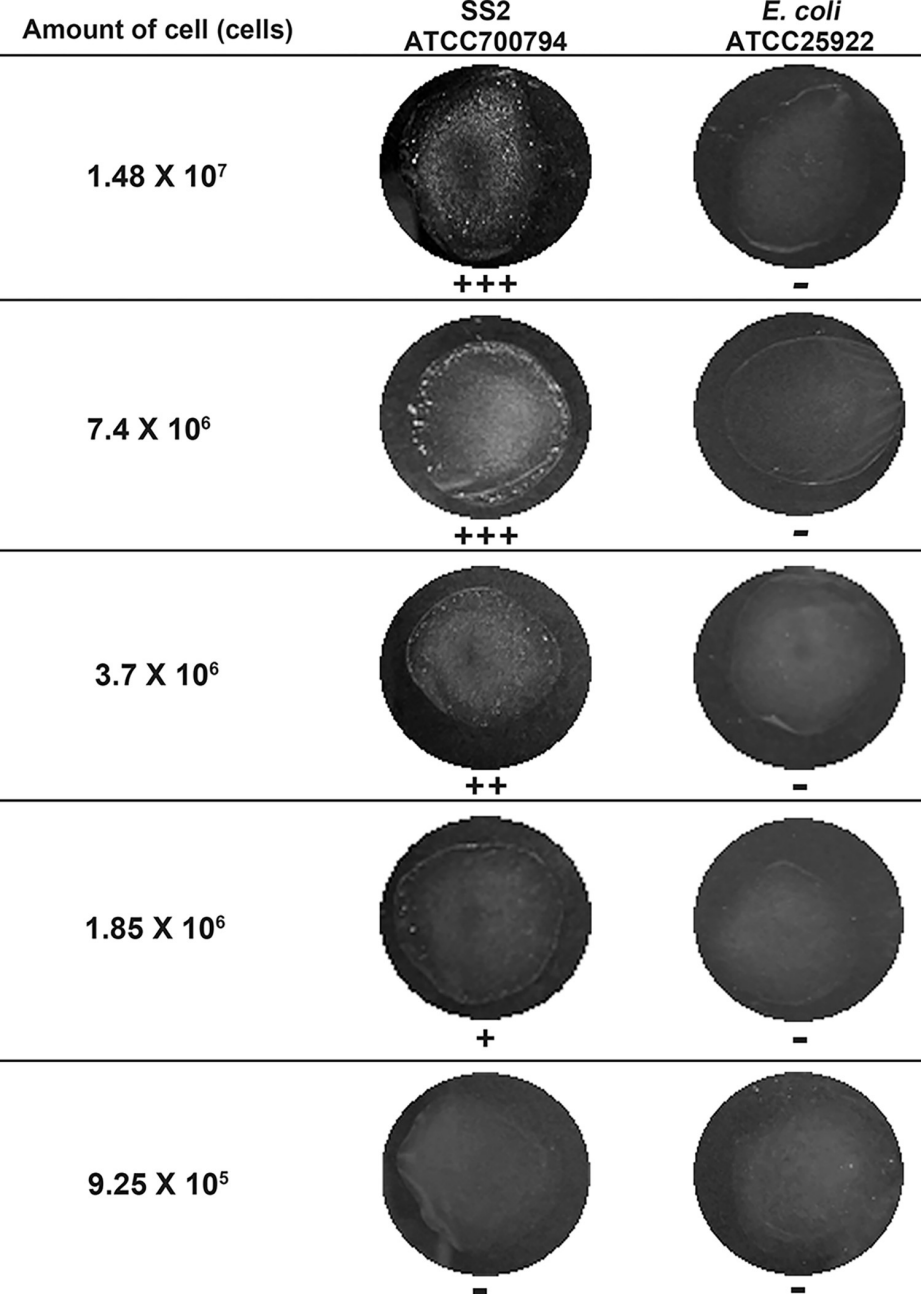
Representative images showing the agglutination reactivity of 47B3 VH-coated beads against different cell densities of SS2, with that for *E*. *coli* cells as a negative control.

Comparison of the sensitivity of the 47B3 VH-coated beads against different concentrations of SS2 cells revealed a positive reaction against as few as 1.85 x 10^6^ cells (Fig 8).

The stability of the 47B3 VH-coated beads was determined by testing them after storage at 4°C with SS2 suspensions. They showed the agglutination (+++) after storage at 4°C for at least 6 months without loss of activity.

## Discussion

Many alternative methods have been developed for the rapid detection of SS2, including immunological and molecular based detection assays [31–34]. The advantages of the LAT over other newly developed methods are that no specific tools are required for the examination, it is simple, inexpensive, and rapid to perform. To develop the test, the conjugation of selected antibodies to latex beads can be performed in several ways, such as adsorption on plain beads or randomly immobilized by cross-linking with functionalized beads [35–37]. However, the selected antibody will be docked onto the bead in a random distribution resulting in a loss in the binding activity [30].

The addition of a free cysteine at the C terminus of VH provides a free thiol (—SH) group, which serves for the site-specific conjugation to maleimide-functionalized particles, forming a stable thioether bond [38]. This strategy was intentionally employed in our study to conjugate the 47B3 VH to the latex beads. Since the VH has one pair of disulfide bonds that play a significant role in the protein folding and binding ability, the 47B3 VH was then selected to be expressed as a soluble protein in *E*. *coli* SHuffle, an engineered strain that can promote disulfide bond formation in its oxidizing cytoplasmic part [39, 40]. The soluble 47B3 VH with an unpaired cysteine at the C-terminus was expressed in the *E*. *coli* cytoplasm in both monomeric and dimeric forms (the latter having a disulfide bridge at the C terminal cysteine), as seen in the non-reducing SDS-PAGE analysis.

After expression and purification, the soluble 47B3VH still retained its bioactivity against SS2. The most serious problem in conjugation via a free cysteine is that reducing the dimeric VH to obtain the homo-monomeric VH can negate its binding ability due to the reduction of the intradomain disulfide bonds. To overcome this, we firstly optimized the VH: DTT molar ratio to obtain VH monomers without interfering with the antigen binding ability. The results indicated that a 1:40 VH: DTT molar ratio was appropriate for the reduction of the 47B3 VH dimer, which was assumed to give approximately one free–SH residue per VH molecule. The 47B3 VH-SH was subsequently conjugated to the maleimide groups on the latex beads in a reaction mixture at pH 6.5–7.5 to spontaneously produce the 47B3 VH-coated beads.

The maleimide linker beads were generated in-house by linking Sulfo-SMCC with NH_2_-polystyrene latex beads of 0.8 μm diameter, since previous reports on LATs demonstrated that the agglutination activity was highest with 0.8-μm diameter latex beads [41].

Once conjugation was achieved, the functionality of the 47B3 VH-coated beads was then verified by combining an equal volume of SS2 cell suspension with 47B3 VH-coated beads on a glass side with a black background. The 47B3 VH-coated beads were able to trigger a strong agglutination reaction with SS2 cells, both the ATCC stain and three clinical isolates. Thus, the optimized VH-bead conjugation described here did not markedly compromise the antigen binding ability of the 47B3 VH antibody. Additionally, the 47B3 VH-coated beads gave a positive reaction with a SS2 cell suspension of only 1.85 x 10^6^ cells, which was slightly less sensitive than that reported before for a LAT based on scFv antibody fragment that gave a positive reaction with 0.23 x 10^6^ cells [35].

The outstanding feature of our LAT that is different from other immunological and molecular based assays is that there is no cross-reactivity with *S*. *suis* serotype 1/2, a serotype that shares a common antigenic epitope with serotype 2 [31, 34, 42]. This is because of the advantage of the VH format that can bind to cryptic epitopes that can differentiate among these subtypes. Another report on the LAT using polyclonal rabbit anti-CPS antibody for detecting SS2 showed cross-reactivity to *S*. *pyogenes* and has not been reported to differentiate between serotypes 2 and ½ [43]. The 47B3 VH-coated beads were stable for at least six months at 4°C, which is broadly similar to that for other LATs based on conventional antibodies that have a stability of 4–12 months at 4°C [44–47].

The LAT described herein could be easily implemented for the identification of suspected S. suis colonies grown on an agar plate in regions where SS2 is highly prevalent. In the further study, the sensitivity and specificity of LAT assay with all *S*. *suis* serotypes and related bacteria will be tested before it can be proposed as a reliable diagnostic tool.

## Supporting information

S1 Raw images Raw image of gel data shown in Figs 1 and 4. (PDF)

S1 Table Raw data of Fig 5. (PDF)
