# Supplementary material for: Development of a latex agglutination test based on VH antibody fragment for detection of Streptococcus suis serotype 2
Source: PLoS One. 2024 Apr 3;19(4):e0299691. doi: 10.1371/journal.pone.0299691 (PMC10990187; doi:10.1371/journal.pone.0299691)
Supplement: S1 Raw images — (PDF) [file pone.0299691.s001.pdf]

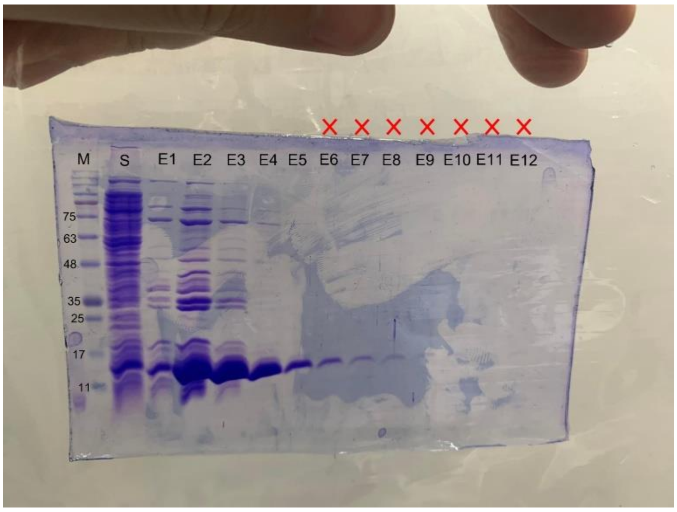

S1 Fig. Raw image of gel SDS-PAGE data shown in Fig 1. SDS-PAGE analysis showing the purification of the recombinant soluble 47B3 VH protein. M: Marker; S: soluble 47B3 VH in the supernatant before purification; E1-E12: elution fractions 1 to 12 of soluble 47B3 VH after purification. This image was captured by the phone's camera.

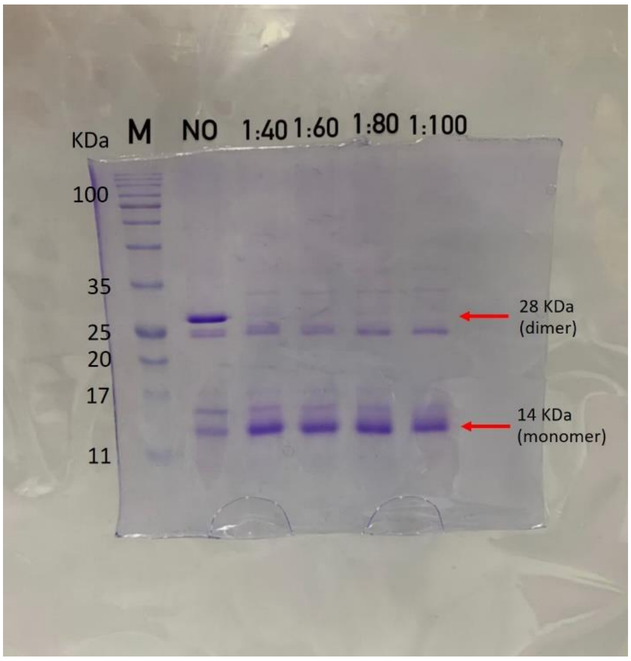

S2 Fig. Raw image of gel SDS-PAGE data shown in Fig 4. The non-reducing SDS-PAGE analysis showing the dimer and monomer forms of 47B3 VH after reduction with different VH: DTT molar ratios. Lane 1: protein MW marker; Lane 2: non-reduced soluble VH fraction; Lanes 3–6: soluble 47B3 VH fraction reduced with VH: DTT molar ratios of 1:40, 1:60, 1:80, and 1:100, respectively. This image was captured by the phone's camera.
