# Supplementary material for: Development of a latex agglutination test based on VH antibody fragment for detection of Streptococcus suis serotype 2
Source: PLoS One. 2024 Apr 3;19(4):e0299691. doi: 10.1371/journal.pone.0299691 (PMC10990187; doi:10.1371/journal.pone.0299691)
Supplement: S1 Table — (PDF) [file pone.0299691.s002.pdf]

S1 Table. Raw data of Fig 5.

VH: DTT	A_{450}			Average	STDEV
non-reduced	1.270	1.292	1.285	1.3	0.01
1:40	1.216	1.238	1.236	1.2	0.01
1:60	1.179	1.083	1.110	1.1	0.05
